# KA-SB: from data integration to large scale reasoning

**DOI:** 10.1186/1471-2105-10-S10-S5

**Published:** 2009-10-01

**Authors:** María del Mar Roldán-García, Ismael Navas-Delgado, Amine Kerzazi, Othmane Chniber, Joaquín Molina-Castro, José F Aldana-Montes

**Affiliations:** Computer Languages and Computing Science Department, Higher Technical School of Computer Science Engineering, University of Málaga, Málaga, 29071 Spain

**Keywords:** Data Service, Query Language, Domain Ontology, User Query, Reasoning Capability

## Abstract

**Background:**

The analysis of information in the biological domain is usually focused on the analysis of data from single on-line data sources. Unfortunately, studying a biological process requires having access to disperse, heterogeneous, autonomous data sources. In this context, an analysis of the information is not possible without the integration of such data.

**Methods:**

KA-SB is a querying and analysis system for final users based on combining a data integration solution with a reasoner. Thus, the tool has been created with a process divided into two steps: 1) KOMF, the Khaos Ontology-based Mediator Framework, is used to retrieve information from heterogeneous and distributed databases; 2) the integrated information is crystallized in a (persistent and high performance) reasoner (DBOWL). This information could be further analyzed later (by means of querying and reasoning).

**Results:**

In this paper we present a novel system that combines the use of a mediation system with the reasoning capabilities of a large scale reasoner to provide a way of finding new knowledge and of analyzing the integrated information from different databases, which is retrieved as a set of ontology instances. This tool uses a graphical query interface to build user queries easily, which shows a graphical representation of the ontology and allows users o build queries by clicking on the ontology concepts.

**Conclusion:**

These kinds of systems (based on KOMF) will provide users with very large amounts of information (interpreted as ontology instances once retrieved), which cannot be managed using traditional main memory-based reasoners. We propose a process for creating persistent and scalable knowledgebases from sets of OWL instances obtained by integrating heterogeneous data sources with KOMF. This process has been applied to develop a demo tool http://khaos.uma.es/KA-SB, which uses the BioPax Level 3 ontology as the integration schema, and integrates UNIPROT, KEGG, CHEBI, BRENDA and SABIORK databases.

## Background

The need for data integration started when the number of applications and data repositories began to grow rapidly. The first approaches appeared in the 80s, and formed the basis for the research in this area. The evolution continued over mediator based systems, such as AMOS II [1], DISCO [2], TSIMMIS [3] and Garlic [4]. Then, agent technology was used in some systems like InfoSleuth [5] and MOMIS [6]. In recent times, the new technologies appearing have been used in data integration: Extensible Markup Language, XML ((MIX [7]), and ontologies (OBSERVER [8]).

The rapid growth of the Internet has provided users with access to an unprecedented number of heterogeneous information sources. This huge amount of information and the complexities of handling it have given rise to a lot of research concerning practical approaches to the Semantic Web.

Semantic Web searches have been based on existing systems, and the proposed approaches offer a limited amount of information for agents. Search engines cannot interpret all the information available because many documents have not yet been semantically annotated. We propose the use of an ontology-based mediator framework (the Khaos Ontology-based Mediator Framework, KOMF) to access varied information from diverse biological databases [9]. KOMF has been successfully instantiated in the context of Molecular Biology for integrating data sources [10].

This application can be used to extract integrated information from the set of databases included in the system, information which is retrieved as a set of ontology instances. However, the analysis of these instances is still limited in KOMF. In order to apply analysis tools it is necessary to store the instances appropriately to facilitate their access. However, the sheer number of instances that must be retrieved make the use of a traditional reasoner unfeasible [11, 12]. Thus, we propose the use of DBOWL [13], a persistent and scalable reasoner that is able to deal with this large number of instances. It stores the ontologies in a relational database, using a description logic reasoner to pre-compute the class and property hierarchies, and to obtain all the ontology information (i.e. properties domain and range), which is also stored in the database. Furthermore, a simple but expressive query language has been implemented, which allows us to query and reason on these ontologies. This reasoner implements both Tbox (ontology structure) queries and Abox (ontology instances) inferences. Tbox queries can be evaluated directly using the query language. Abox inferences however are evaluated when a query is sent to the system to obtain complete results. Both Tbox queries and Abox inferences are implemented using only the information stored in the database.

In summary, the goal of this paper is to present a user query system based on combining a data integration solution with a reasoner, to boost the analysis potential for the knowledge obtained in response to user queries. The combination of a data integration system with a reasoner is a novel approach that opens up new ways of analyzing the information based on the knowledge. This is also the way to obtain a mediator which can reason on the integrated knowledge.

This process has been used to implement a demo tool http://khaos.uma.es/KA-SB showing how the BioPax Level 3 ontology can be used as the integration schema to integrate UNIPROT [14], KEGG [15], CHEBI [16], BRENDA [17] and SABIORK [18] databases.

### Previous works

This section describes the two previous works on which the proposal is based. First, we will show the main features of KOMF and how it can be configured to integrate biological data. Then, we will describe DBOWL, a persistent and scalable reasoner.

#### • KOMF

**Figure 1:**
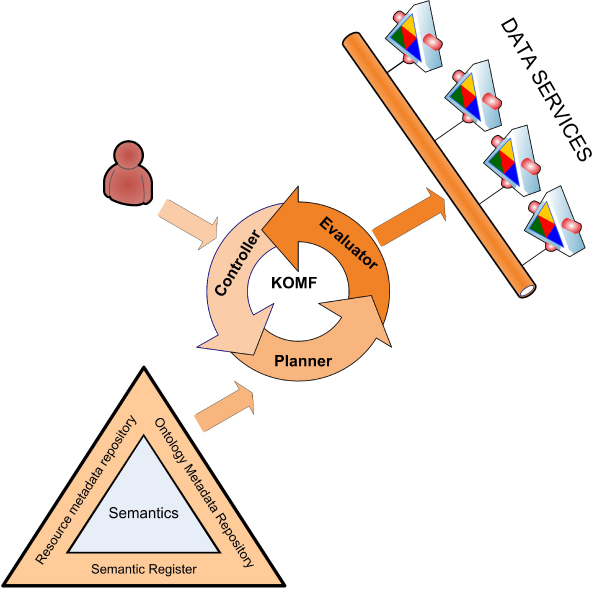
**KOMF architecture**. This mediator is based on the use of ontologies to integrate heterogeneous data through data services.

In this section, we briefly describe an ontology-based mediator framework (KOMF) which uses a Semantic Directory (SD-Core [19]), a generic infrastructure to register and manage ontologies, their relationships and also information relating to the resources. In the proposed framework (Figure 1) our goal is to provide access to the data using a common data model, and a common query language. Our architecture provides a semantically coherent model representation of the combined data from the wrapped data sources and transparent access to the combined data through queries to the mediating view.

In this context, wrappers are an important part of the internal elements of Data Services [20]. A wrapper accepts queries from the mediator, translates the query into an appropriate query for the individual source, performs any additional processing and returns the results to the mediator. Data sources in some domains such as Molecular Biology are usually public and downloadable. For these cases we have designed patterns to retrieve data sources stored as flat files for later storage in an XML database. Data Services, independently of the development process, are distributed software applications that receive queries in XQuery and return XML documents.

As the proposal is to use ontologies as schemas to integrate data, we have chosen a Global as View (GAV) approach [21]. In GAV, each source is related to the global schema (ontology in our case) by means of mappings. Moreover, the use of ontologies will allow us to take advantage of reasoning mechanisms to improve the query rewriting. The KOMF architecture is composed of three main components: *the Controller, the Query Planner and the Evaluator/Integrator*.

#### • DBOWL

DBOWL [13] is a persistent and scalable OWL (Web Ontology Language) reasoner. DBOWL stores the OWL-DL ontologies in a relational database, and supports Tbox queries (queries on the ontology structure), Abox inferences (reasoning on the ontology instances) and Extended Conjunctive Queries (ECQ) queries [22]. Currently we are finishing a SPARQL [23] (SPARQL Protocol and RDF Query Language) query engine for DBOWL (neither DBOWL nor the query engines have been released yet). In order to create the relational database for ontology storage, a Description Logic Reasoner is used. Thus, the consistency of the ontology as well as the inferences about the ontology structure are delegated to this reasoner and DBOWL focuses on reasoning on instances (large numbers of them). Both, Tbox queries and ECQ queries are implemented by translation to SQL. Abox inferences are implemented by java functions and SQL views.

**Figure 2:**
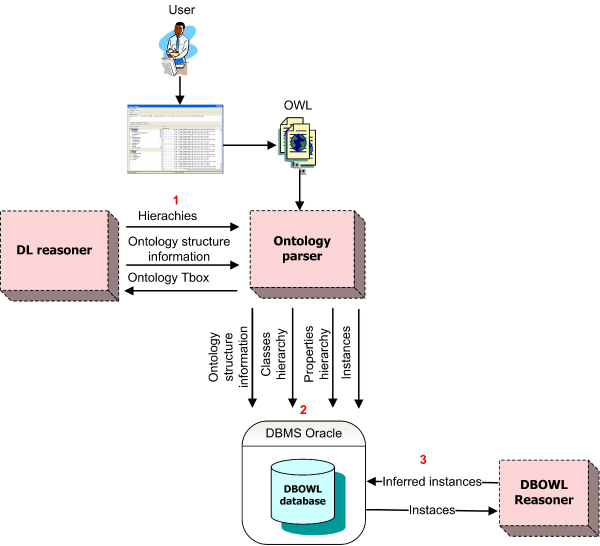
**DBOWL Storage System**. DBOWL Storage System.

DBOWL consists of two services, an OWL storage system and an OWL querying system. The OWL storage system (Figure 2) stores the OWL ontology in the database. The relational schema is implemented using the Oracle database management system and all the necessary information for implementing Tbox queries and Abox inferences is then stored in the database. Finally, the DBOWL reasoner evaluates the java functions implementing the Abox inference and creates the SQL views containing the inferred instances.

DBOWL implements both Tbox queries and Abox inference. Tbox queries can be evaluated directly using the query language. On the other hand, Abox inferences are evaluated when a query is sent to the system to obtain complete results. Currently, DBOWL supports all the Tbox queries implemented by RACER [11]. In order to implement them, the information obtained from the DL reasoner is stored in the corresponding tables at load time. The Abox inference rules currently supported by DBOWL cover OWL-DL completely.

In order to demonstrate the performance of DBOWL, we use UOB (University Ontology Benchmark) [24], a well known benchmark to compare repositories in the Semantic Web. This benchmark is intended to evaluate the performance of OWL repositories with respect to extensional queries over a large data set that commits to a single realistic ontology. Furthermore, the benchmark evaluates the system completeness and soundness with respect to the queries defined. This first experiment is conducted on a PC with Intel Quad Core of 2.3 GHz and 3 GB memory, running Windows Vista with Java JRE 1.6.0.7. We use the benchmark 20 MG and 100 MG ontologies, which contain around 200.000 and 1.000.000 individuals respectively. DBOWL response times are quite good and DBOWL also returns all expected results.

## Methods

In this section we describe a process for creating a persistent and scalable knowledgebase from integrated data. As described in the previous section, users can use KOMF to query heterogeneous data sources, and use this information to perform domain specific analysis. However, KOMF has limited reasoning capabilities. Therefore, the proposed methodology introduces DBOWL as a persistent reasoner to perform more complex analysis.

**Figure 3:**
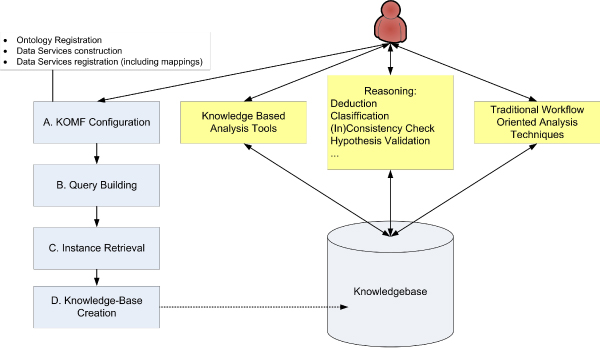
**KA-SB, tool information flow**. KA-SB, tool information flow.

Thus, the designed methodology establishes a set of operations to be performed when a knowledgebase is to be constructed from diverse data sources (Figure 3). It follows four steps:

A. KOMF configuration (A in Figure 3). This task aims to produce the necessary elements to integrate information from heterogeneous data sources. It involves firstly registering the domain ontology to represent the domain. The next step is to create the necessary data services, register them in the system and then set up the relationships between each data service schema and our domain ontology. After this configuration, users can send queries in terms of the domain ontology, which will be solved using the registered data services. This part requires a lot of work that remains mainly in the data service development and mapping definition (when using an existing ontology), as has been described in a previous section.
B. Query building (B in Figure 3). As we aim to produce a knowledgebase centered on a specific need, it is necessary to design a query (or a set of queries) to retrieve all the information that will be later analyzed. This step could be done using The Visual Semantic Browser (VSB [25]), which allows users to browse an ontology and query KOMF to retrieve relevant information. VSB is a tool designed to visualize different views of semantics. The main aim of the tool is to enable user interaction, also to locate and use semantics usually only available to computers. It provides the necessary elements to facilitate the inclusion of new algorithms with little effort. Also, some algorithms have been adapted in this prototype for the visualization of ontology groups, mappings, ontologies and instances.
C. Instance retrieval (C in Figure 3). The designed query is executed using KOMF, obtaining a set of instances as RDF (Resource Description Framework) documents.
D. Knowledgebase creation (D in Figure 3). The domain ontology and the retrieved RDF documents (for which the user requires a more sophisticated analysis) are used to generate the query-based knowledgebase using DBOWL.

**Figure 4:**
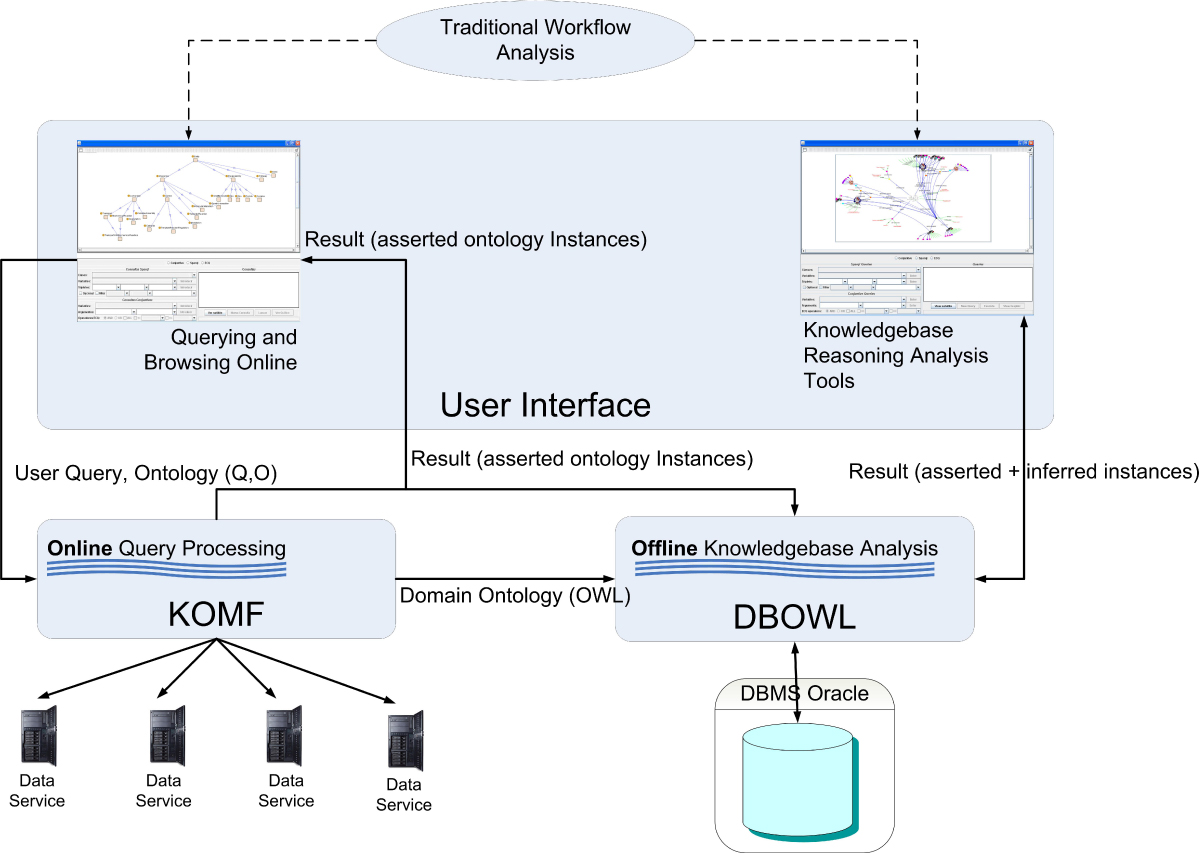
**KA-SB structure**. The methodology is based on the use of KOMF to retrieve information as ontology instances. When a user retrieves information that needs further analysis, the tool allows him/her to create a persistent knowledgebase. This knowledgebase could be used to perform more detailed and complex analysis over a specific set of information.

The methodology requires the use of the KOMF framework and the DBOWL reasoner described previously (Figure 4). User queries are sent to KOMF (see [9] for more details about the data service creation and mapping description in KOMF) to retrieve the required instances (those necessary for more sophisticated analysis), which will be stored in DBOWL (D in Figure 3). Then, analysis tools can take advantage of the reasoning capabilities of DBOWL. Both user interfaces can publish their programming interface so that they can be used in traditional life science workflows as another data source or data transformation tool.

**Figure 5:**
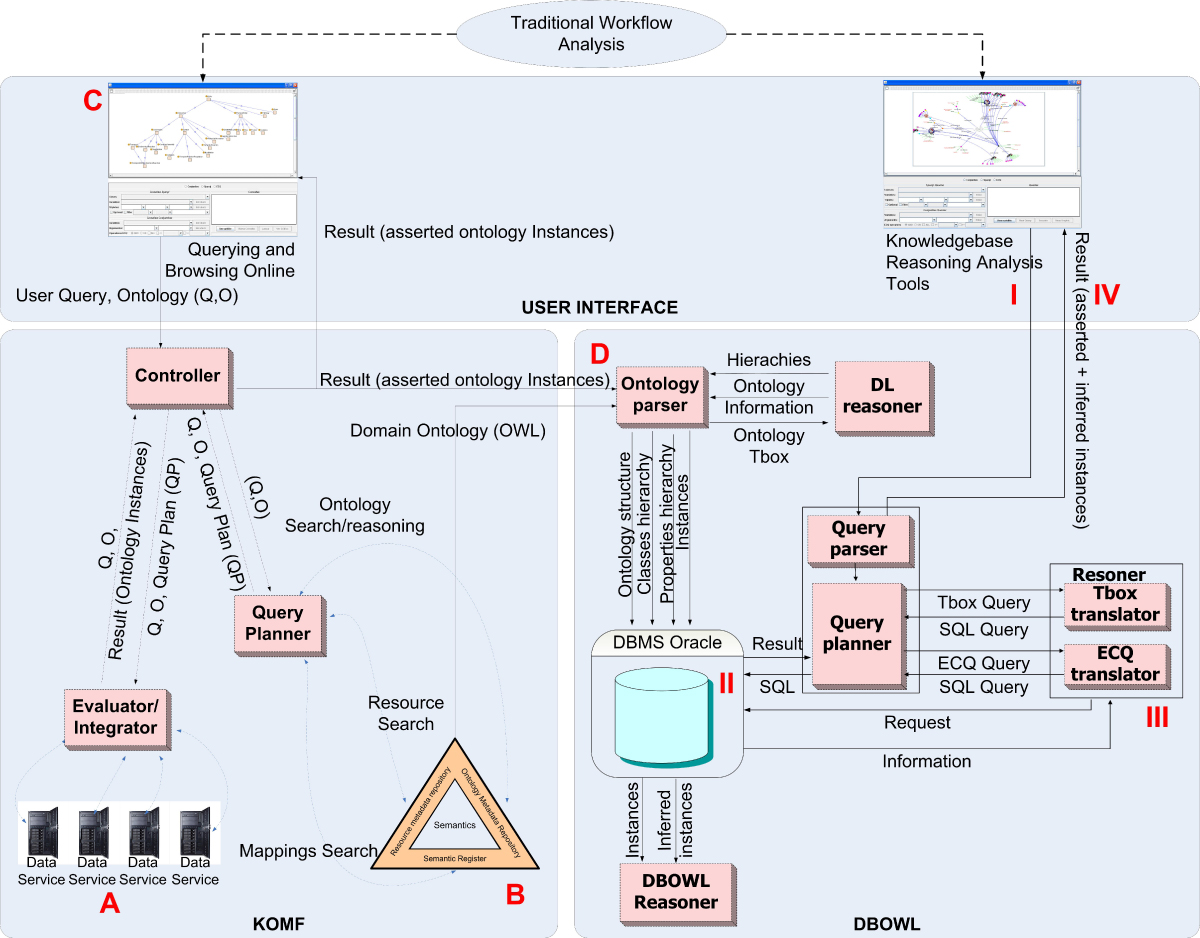
**KA-SB implementation details**. The internal elements of KOMF allow users to perform online queries, while DBOWL provides a persistent reasoner to perform more complex analysis over specific sets of information.

The proposed methodology has been used to produce a demo tool (Figure 5) for accessing biological data and to allow users to create knowledgebases from retrieved data, enabling its subsequent analysis using reasoning. In order to show how the use case is built we will describe each step as described in the methodology.

### KOMF configuration

**Figure 6:**
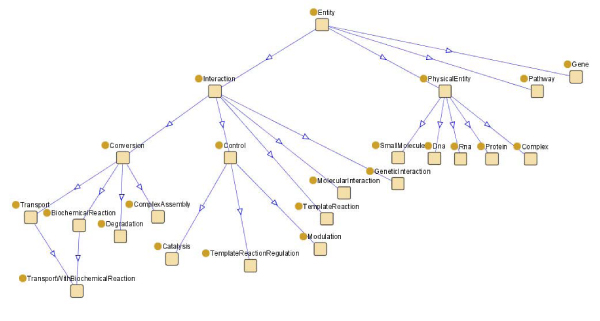
**Part of the ontology BioPax Level 3**. This ontology has been registered for integrated access to biological data in this use case.

In order to configure KOMF we need to carry out the following tasks:

1. Develop a set of Data Services. In this use case we have developed several data services for accessing metabolic data: UNIPROT [14], KEGG [15], CHEBI [16], BRENDA [17] and SABIORK [18] (A in Figure 5).
2. Choose a domain ontology as the integration schema of KOMF. In this use case we have chosen BioPax Level 3 http://www.biopax.org/, which covers metabolic pathways, molecular interactions, signaling pathways (including molecular states and generics), gene regulation and genetic interactions. Figure 6 shows the entities part of this ontology. It has been registered in SD-Core (B in Figure 5).
3. The data services developed are also registered in SD-Core, by defining the mappings between the data service schemas and the domain ontology (B in Figure 5).
4. Finally, KOMF can be queried to obtain integrated results from the registered data services.

### Query building

**Figure 7:**
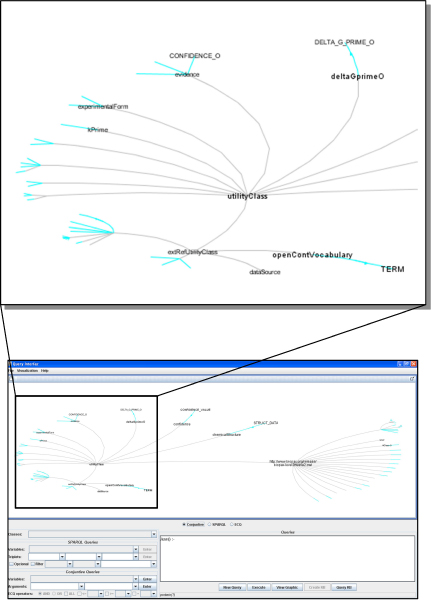
**Query Interface**. This part of the tool enables building user queries easily.

In order to enable users to query KOMF (C in Figure 5) we have developed a tool that uses an extension of VSB [25], which provides a user interface for visualizing the registered ontology and creating the user query (see Figure 7). The interface allows users to select concepts of the ontology to build the queries easily. Thus, this interface uses a heuristic to suggest links between predicates using the variables to facilitate the user query.

**Figure 8:**
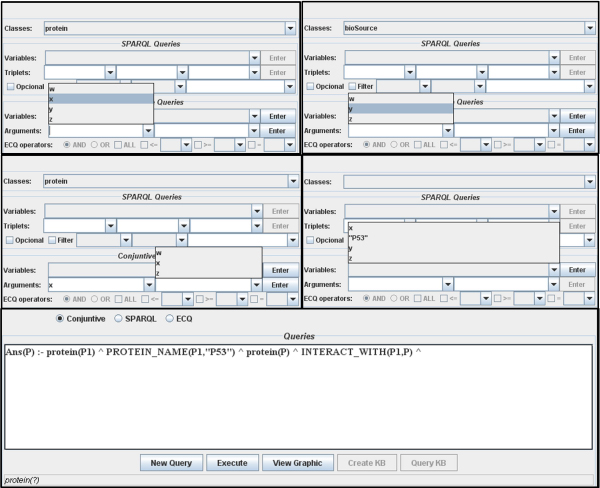
**Step by step query building**. The user selects the name and organism of the target protein, and then introduces the predicates to search for interacting proteins.

For example, (Figure 8) in the domain ontology we have the concept *Protein* and *Organism* (linked through the object property *bioSource*):

- When the user clicks on the Protein concept, the tool proposes to introduce the predicate *Protein (X)*;
- When the user clicks on the Organism concept, the tool asks users to input the predicate *Organism (Y)*;
- If the user clicks on the property bioSource, the tool proposes the predicate *bioSource (X, Y)*.
- If the user clicks on the Protein concept, the tool asks users to input the predicate *Protein (Z)*;
- Finally, if the user selects the property Interact_with, the tool will propose to use *Interact_with(X, Z)*;

Using this user query interface, users can query KOMF to retrieve useful information. In order to show the use of this interface we will describe some simple examples that will be further detailed in later sections.

### Instance retrieval

**Figure 9:**
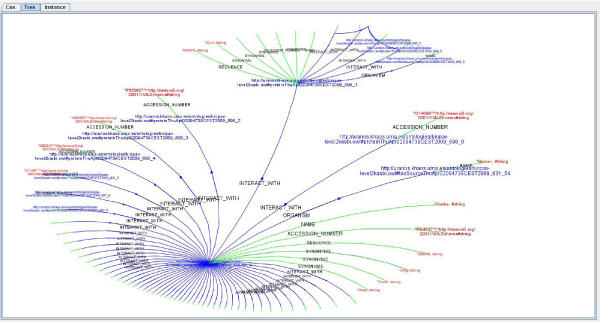
**Instance visualization**. Results obtained from the mediator can be visualized as RDF instances, flat files and a graphical representation.

The user query is evaluated using the configured KOMF, the query is planned and the result is obtained as a set of instances (Figure 9). These instances can also be visualized using the user interface. In this sense, the results obtained from the mediator can be visualized as RDF instances, flat files and a graphical representation. Thus, expert users can directly analyse RDF documents, while other users can take advantage of an easy to interpret graph, showing the instances and their relationships.

For the query example shown in the previous section, the user will obtain a set of proteins interacting with the target ontology. Thus, the user can easily visualize the interaction network of this protein in a graphical way.

However, at this point the advantage of using semantics is limited to the explicit representation of certain knowledge. We may need to take advantage of reasoning to discover new knowledge from the retrieved data, as will be shown in the following section.

### Knowledgebase creation and knowledge based analysis

Using the set of retrieved instances, the user can decide to make other queries on the mediator, but he/she can also decide to make this knowledge permanent in the knowledgebase, and can take advantage of the DBOWL reasoner.

In this demo tool each user can have five sets of instances permanently stored in DBOWL, where they can be analyzed and reasoned on. Once the knowledgebase has been created the users can use it to perform different analyses using analysis tools. For example, the visualization tool, VSB, can be used to analyze the structure of the knowledge stored. This visualization tool can be configured to use different icons for different instance types, so end users can better understand the resulting graph.

Furthermore, new tools can be developed or existing tools can be adapted to analyze specific issues based on the expertise of domain experts. The advantage of using DBOWL is that these tools (I in Figure 6) can take advantage of a persistent storage (II in Figure 6) and reasoning to infer new knowledge (III in Figure 6). Thus, results (IV in Figure 6) can contain asserted instances plus those obtained through reasoning.

### Reasoning examples

In this section some theoretical examples are shown, which use a knowledgebase with useful information for systems biology researchers taking advantage of the tool described.

The retrieval of information about different Pathways will provide the user with a set of interactions. These interactions are represented as instances of the Interaction concept or any of its descendants. Thus, they can be classified using these descendants. For example we can have different Control interactions, which can be classified as *Catalysis*, *Modulation* or *TemplateReactionRegulation*. These interactions have a *controlType* property that can take values such as: INHIBITION, ACTIVATION, INHIBITION-ALLOSTERIC, INHIBITION-COMPETITIVE, etc. However Catalysis can only be of type ACTIVATION, and so it has been defined in the ontology as a functional property (it can only take one value for this property) and has at least one value for controlType property with value activation:

- controlType is Functional
- ∃ controlType has "ACTIVATION"

However, once the knowledgebase is created the set of instances may contain errors. The use of the reasoner will solve this problem. If an interaction is retrieved from the mediator, it is classified as Catalysis. However, if the control type is "INHIBITION", the reasoner infers that this is an inconsistency in the ontology. For example, the interaction named *'AMP [cytosol] negatively regulates Phosphorylation of ChREBP at Thr(666) by AMP kinase*' has a control type inhibition, and so its classification as a Catalysis will be resolved as an annotation error.

Another example is the physical entity, where we can find that an entity P is an instance of Protein and Complex classes (in two different databases). In this case the reasoner also infers that the knowledgebase has inconsistencies (Protein and Complex are defined as disjoint classes in this ontology). For example the protein complex "Cytochrome b6f Complex" may be annotated in one database as a Protein and as Complex in a different database. Thus, this inconsistency will be detected by the reasoner, and the application using this information can act to resolve this inconsistency.

## Results

In this paper we have presented a novel system that combines the use of a mediation system (KOMF) with the reasoning capabilities of a large scale reasoner (DBOWL) to provide a way of finding new knowledge.

The study of data integration systems has allowed us to determine their main elements, and thus to extract the pattern for building this kind of system.

HERMES [26], DISCO [2] and TSIMMIS [3] are well known mediator systems. Essentially, all of these tools have a three-tier software architecture: Clients connect to a *mediator* [27]. The mediator parses a query, carries out query rewrite and query optimization, and executes some of the operations of a query. The mediator also maintains a catalog to store the *global schema* of the whole heterogeneous database system (i.e., the schema used in queries by application programs and users), the *external schema* of the component databases, and statistics for query optimization.

In the specific field of biological data the following examples exist: TAMBIS [28], BioDataServer [29], KIND [30], BioZoom [31], BioKleisli [32], DiscoveryLink [33], BioBroker [34] and BioMoby [35].

DiscoveryLink [33] is one such system, targeted to applications from the life sciences industry. It provides users with a virtual database to which they can pose arbitrarily complex queries, even though the actual data needed to answer the query may originate from several different sources, and no individual source, by itself, is capable of answering the query.

VIRTUOSO [36], comprehensive data integration software developed by OpenLink Software, is also capable of processing distributed queries. Because Virtuoso is also a native quad store, its strength is its scalability and performance. In addition to the commercial edition, an open source version is also available. A relatively new application also provided by OpenLink is the *OpenLink Data Spaces* platform, which is said to be able to integrate numerous heterogeneous data from distributed endpoints.

OBSERVER [8] presents an approach which aims to enhance the scalability of query processing in a global information system. Besides, users can express queries using semantic concepts. This approach makes use of pre-existing ontologies to integrate the underlying data sources. Thus, repositories can be viewed with respect to relevant semantic concepts. Semantic relationships can be defined between different ontologies, and they can be used to solve user queries. Information loss is also dealt with to provide a fast response to users when exact results are not required.

Model-Based Mediation [37] is a paradigm for data integration in which data sources can be integrated, using auxiliary expert knowledge. This knowledge includes information about the domain and is the glue that binds data source schemas together. The expert knowledge is captured in a data structure called Knowledge Map. In Model-Based Mediation, the mediation architecture is extended, carrying data sources from the data level without semantics to the conceptual model level. This architecture introduces semantics into data sources and mediators, but it is not published nor is it accessible to agents or applications. Mediators are monolithic systems and they are strongly coupled to wrappers, limiting dynamic integration and interoperability.

DBOWL is an OWL reasoner. As OWL is based on DL, we must study DL reasoners. Of these, RACER [11] is the most relevant and one of the most complete, and it implements both Tbox and Abox reasoning. Furthermore, it provides its own query language, which allows simple conjunctive queries to be evaluated. It is not persistent however, and reasoning is implemented by reducing it to satisfiability. This means on the one hand, that each time we use the reasoner, we must load and process the ontology and, on the other hand, that large ontologies (with a large number of instances) cannot be loaded. Finally, RACER is currently a commercial tool, and therefore, other DL reasoners, like PELLET [12] are becoming more popular. PELLET provides the same functionality as RACER but also has the same problems. In the past few years there has been a growing interest in the development of systems for storing large amounts of knowledge in the Semantic Web. Firstly, these systems were oriented to RDF storage [38–40]. Nowadays, research is oriented to massive OWL storage. Several alternative approaches using relational technology have been presented. Instance Store [41] uses a DL reasoner for inferring Tbox information and storing it in a relational database. However, the ontology definition language does not allow the definition of binary relationships. From our point of view, this is an important expressiveness limitation. Moreover, Instance Store only evaluates some Abox reasoning, namely subsumption of concepts and equivalent classes. It implements them by reducing them to terminological reasonings and evaluates them using a DL reasoner. On the other hand, the QuONTO [42] system reduces the ontology definition language to DL-Lite [43], a description logic which is a subset of OWL-DL. Therefore, the soundness and completeness of the reasonings is ensured. It evaluates subsumption of concepts Abox reasoning and conjunctive queries. The queries are rewritten using the Tbox information and are translated to SQL. DLDB-OWL [44] extends a relational database with OWL inferences. This proposal uses a DL reasoner as Instance Store but the database schema is more complex. In its public distribution only the subsumption of concepts is implemented, but it is implemented using only the information stored in the database. Finally, Minerva [45] also stores the ontology in a relational database, but uses a DL reasoner for evaluating Tbox reasonings and a rule engine to evaluate Abox reasonings which are defined using Description Logic Programs (DLP [46]) rules partially covering OWL-DL. Minerva combines relational technology with logic rules and also evaluates SPARQL queries. Our proposal aims to combine all these results, providing a persistent and scalable tool for querying and reasoning on OWL ontologies. To do this, we provide an optimized storage model which is efficient and scalable, we implement reasoning on top of a relational database and combine reasoning and querying.

The presented tool uses a graphical query interface to build user queries easily. This interface shows a graphical representation of the ontology and allows users to build queries by clicking on the ontology concepts. Protégé [47] is a well known editor for ontologies that has been applied to edit OWL ontologies. However, Protègè provides an interface oriented to semantic web experts, and so it does not provide an easy-to-use query interface for final users.

However, the proposed system can be improved in many ways. The main drawback of this proposal is the configuration of KA-SB, which because it requires performing some manual steps, it is difficult for non-expert users:

- Firstly, the development (or search) of a domain ontology is necessary. This is an important issue in all the proposals for using semantics to improve any kind of process. This could be solved by designing new tools for scientists who are not semantic Web experts. In this sense, some systems have been proposed [48].
- Secondly, the configuration of KOMF requires the development of some steps that cannot be achieved by non-expert users. The first difficulty is that for accessing the information it is necessary to implement data services. This problem has several solutions: either to develop automatic tools for creating a data service (there are some proposals for this such as [20]) or to develop a repository with available data services. In line with this last solution we are working on making public all the developed data services in the biological domain. The second difficulty is that these data services have to be registered in the mediator. This issue can be solved by including automatic tools to find the mappings between the domain ontology and each data service schema (currently the matching tools are very accurate [49]).

## Conclusion

The combination of data integration solutions with reasoners to provide analysis tools in biology is a novel approach that opens up new possibilities in domains such as Systems Biology. The process described by KA-SB shows a way to use retrieved instances from user queries in a reasoner. This process has been applied to develop a demo tool http://khaos.uma.es/KA-SB, which provides a lot of opportunities to take advantage of the integrated information by means of a user interface for testing different queries. The reasoner allows users to exploit the results to search for new knowledge or to perform analyses. However, in a context like Systems Biology it is important to provide tools able to deal with a large amount of information, as the OMIC era has produced an enormous amount of freely available data.

Our approach can be useful for real Systems Biology applications, especially for those aiming to provide end-user interfaces with extended capabilities. In this sense, we will study how to apply our proposal to extend a real application in Systems Biology (developed using KOMF), the System Biology Metabolic Modelling Assistant [50], which is a tool developed to search, visualize, manipulate and annotate identity data and assist in annotating the kinetic data.

## List of abbreviation used

KOMF: Khaos Ontology-based Mediator Framework
XML: Extensible Markup Language
SD-Core: Semantic Directory Core
GAV: Global as view
OWL: Ontology Web Language
ECQ: Extended Conjunctive Queries
SPARQL: SPARQL Protocol and RDF Query Language
UOB: University Ontology Benchmark
VSB: Visual Semantic Browser
RDF: Resource Description Framework
DLP: Description Logic Programs
